# Signatures of tumor microenvironment-related genes and long noncoding RNAs predict poor prognosis in osteosarcoma

**DOI:** 10.1371/journal.pone.0326876

**Published:** 2025-07-16

**Authors:** Ying Lu, Li Zhou, Zhanyu Yang

**Affiliations:** 1 Department of Orthopaedics, Hunan Provincial People’s Hospital (the First Affiliated Hospital of Hunan Normal University), Changsha, Hunan, China; 2 Hunan Emergency Center, Changsha, Hunan, China; 3 Department of Orthopaedics and Traumatology, Nanfang Hospital, Southern Medical University, Guangzhou, Guangdong, China; Universita degli Studi della Campania Luigi Vanvitelli, ITALY

## Abstract

**Introduction:**

Osteosarcoma is an aggressive bone cancer with poor outcomes, especially in young individuals. This study sought to identify tumor microenvironment-related genes (TMIEGs) and associated long noncoding RNAs (TMIELs) that influence patient prognosis.

**Materials and methods:**

Data from the TARGET osteosarcoma and GTEx muscle datasets were analysed to calculate stromal and immune scores, dividing patients into high- and low-score groups. Differential gene expression was assessed, and prognostic TMIELs and TMIEGs were identified through regression analyses. Prognostic signatures were evaluated via Kaplan‒Meier curves, receiver operating characteristic (ROC) analysis, and Cox regression, while immune cell composition was analysed via CIBERSORT.

**Results:**

Three prognostic TMIELs (AC090559.1, LINC01549, SENCR) and three TMIEGs (DOK2, RHBDL2, NPW) were identified. High-risk patients have poorer survival outcomes, with immune processes possibly reducing the risk of osteosarcoma. Prognostic signatures effectively predict overall survival.

**Conclusion:**

TMIEGs and TMIELs can reliably predict survival in patients with osteosarcoma, suggesting their potential as therapeutic biomarkers and the need for further research.

## Introduction

Osteosarcoma is a nonhematopoietic primary malignant tumor of the bone that predominantly affects children, adolescents, and young adults. Although it represents less than 1% of all cancers according to the Surveillance, Epidemiology, and End Results (SEER) Program in the United States [1], it is characterized by a disproportionate distribution and poor outcomes. Typically, osteosarcoma originates in the metaphysis of long bones, most frequently occurring in the humerus, proximal tibia, and distal femur [2]. However, axial tumors are more invasive and tend to metastasize to the lung [3]. This high incidence and poor prognosis contribute to the high mortality associated with osteosarcoma. In recent years, it has consistently ranked as the second most lethal tumor disease among adolescents and children [4], with an annual incidence increase of 0.3% [5]. Despite the application of multimodal treatment approaches, the 5-year survival rate for nonmetastatic osteosarcoma remains stable at approximately 67%, and even for those with distant metastasis, the 5-year survival rate does not exceed 20% [6,7]. The challenges in managing osteosarcoma are attributed to its distinct characteristics, including recurrence, metastasis, and resistance [3,8]. Furthermore, variability in treatment outcomes can be linked to genetic heterogeneity, resulting in different responses among patients with the same clinical or pathological classification and treatment regimen [9–11].

In the quest to improve outcomes for osteosarcoma patients, there has been a growing exploration of targeted therapies and immunotherapies. These include investigations into the use of tyrosine kinase inhibitors, immune checkpoint inhibitors, and even tumor cell vaccines [12–14]. However, these therapeutic approaches have achieved limited success in significantly enhancing clinical outcomes. This can be attributed to the complex nature of the tumor microenvironment, which is significantly involved in the development of osteosarcoma [15–17]. The tumor microenvironment encompasses the intricate internal milieu where tumor cells thrive and proliferate, driven by intricate interactions mediated by diverse signalling molecules in the extracellular matrix [18,19]. Numerous studies have emphasized the critical role of the tumor microenvironment in the progression of tumors [20,21]. Within this microenvironment, immune cells that infiltrate the tumor can either impede or facilitate tumor progression, influencing diverse aspects of the disease, such as its initiation, development, dissemination, recurrence, and metastasis [22]. Recent efforts in cancer therapy have increasingly focused on harnessing the potential of tumor-infiltrating immune cells within the tumor microenvironment, yielding promising results that hold the potential to significantly improve patient prognosis [23–25]. This emphasis on understanding and manipulating the tumor microenvironment and immune cell interactions offers new hope in the battle against osteosarcoma.

Long noncoding RNAs (lncRNAs), which exceed 200 nucleotides in length, constitute a significant category of noncoding RNAs, making up approximately 80% of all noncoding RNAs [26]. Originally dismissed as transcriptional byproducts lacking protein-coding capacity, lncRNAs were considered genetic “junk” or mere noise [27,28]. Recent research, however, has revealed that lncRNAs play pivotal roles in diverse biological processes. They are involved in gene transcription, chromatin modification, and epigenetic regulation and operate as competing endogenous RNAs (ceRNAs) [29]. As ceRNAs, they function as molecular sponges for specific microRNAs (miRNAs), thus influencing miRNA-driven gene silencing. miRNAs, a type of small noncoding RNA that usually consists of 18–25 nucleotides, bind to mRNA molecules via base pairing, leading to the suppression of translation or the breakdown of mRNA [30]. Furthermore, changes in the expression of lncRNAs play a role in the initiation and progression of osteosarcoma. Certain lncRNAs exhibit elevated expression levels and function as oncogenes, driving tumor growth and spread. In contrast, reduced expression of other lncRNAs has been identified as having a tumor-suppressive effect in osteosarcoma [31]. However, a significant portion of lncRNAs remain uncharacterized concerning their functions in the pathogenesis of osteosarcoma, necessitating further investigation.

Previous studies have established the critical roles of M2 macrophages, monocytes, and CD8 + T cells in shaping the immune microenvironment of osteosarcoma [32]. Within this context, effector memory CD8 + T cells and type 2 T helper cells play vital roles in activating various signalling pathways, thereby regulating tumor cell proliferation, motility, apoptosis, metastatic potential, and responsiveness to chemotherapy [33]. Additionally, patients with osteosarcoma who exhibit increased infiltration of macrophages and B cells tend to have a more favourable prognosis. Consequently, genes related to the tumor microenvironment in osteosarcoma have prognostic value. The potential prognostic genes linked to the microenvironment in osteosarcoma offer promising prospects for tailoring optimal treatment strategies and enhancing current therapeutic regimens [34].

In light of the current limitations in our understanding of osteosarcoma, there is an increasingly urgent imperative to delve into the intricate mechanisms governing its development. This pursuit aims to identify novel and effective prognostic markers, further enabling precise risk assessment and patient management. This approach aligns seamlessly with the tenets of precision medicine. In our study, our primary objectives were to pinpoint prognostic signatures among genes and lncRNAs associated with the tumor microenvironment in osteosarcoma. These hub signatures hold promise for predicting the clinical outcomes of osteosarcoma patients. Additionally, we endeavoured to dissect their intricate interactions and decipher their functional characteristics. Ultimately, we seek to construct models incorporating these tumor microenvironment-related markers, which have the potential to serve as invaluable tools for both prognostics and diagnostics in the context of osteosarcoma.

## Materials and methods

### Data abstraction and preparation

**Fig 1:**
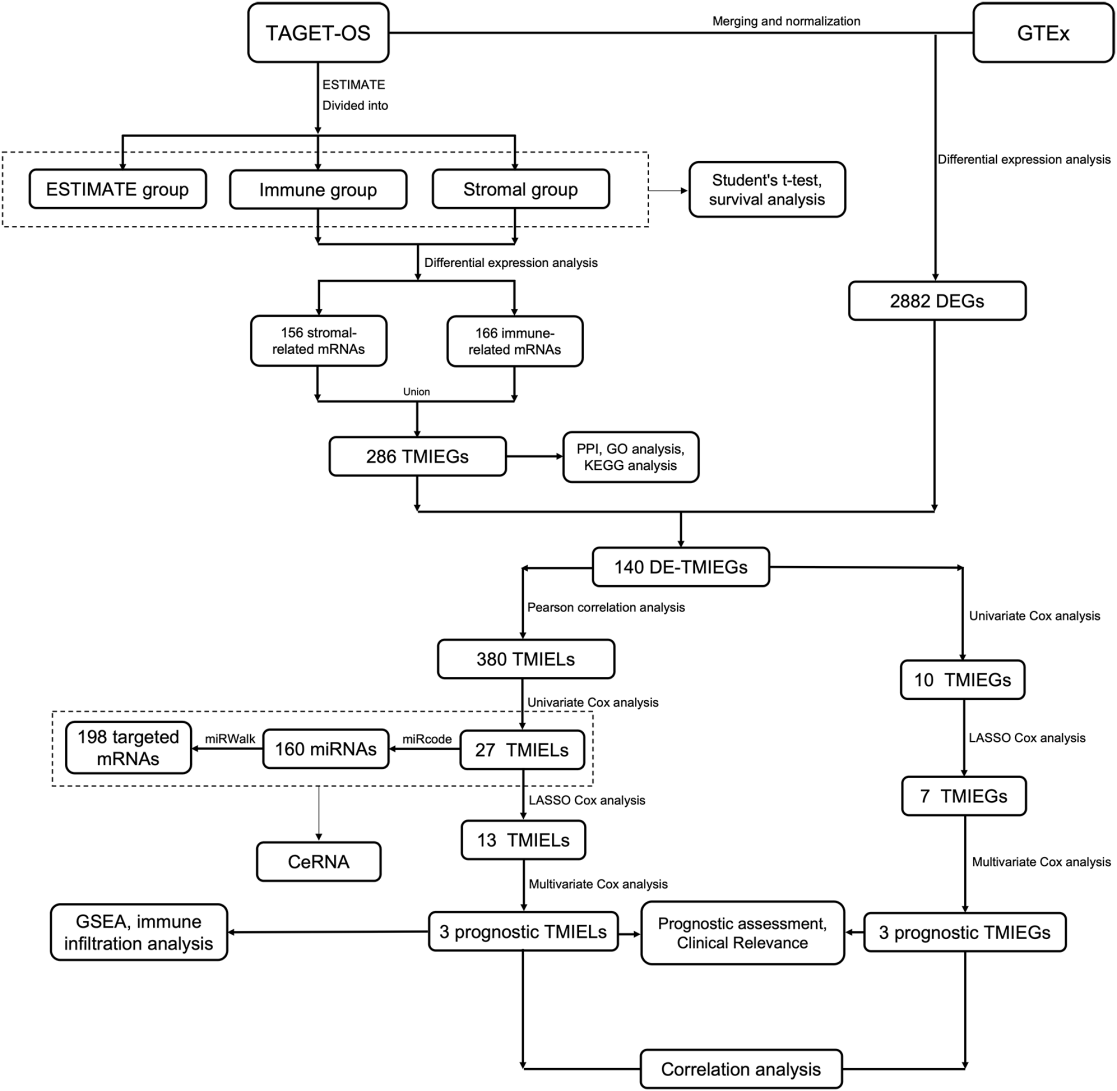
Study design and workflow.

The study design and workflow are depicted in Fig 1. Data on the expression and detailed clinical characteristics of osteosarcoma samples were obtained from the Therapeutically Applicable Research to Generate Effective Treatments database (TARGET) (https://ocg.cancer.gov/programs/target). TARGET is a genomics program committed to employing a comprehensive genomic approach to uncover molecular changes in pediatric cancer development and to facilitate the exploration of more effective and less toxic therapies. Both osteosarcoma tissue and skeletal muscle are derived from mesenchymal tissue of the mesoderm. To provide a comparative baseline, gene expression profiles from normal skeletal muscle samples were retrieved from the Genotype-Tissue Expression database (GTEx) (https://www.gtexportal.org/home). GTEx serves as an open repository containing transcriptome data across various normal human tissues. GSE39055, as an independent dataset, was downloaded from the Gene Expression Omnibus (GEO) database (http://www.ncbi.nlm.nih.gov/geo) for validation of prognostic features. The datasets in our study are publicly available. In the interest of data consistency, we conducted a Log2(x + 1) conversion for values of fragments per kilobase of transcript per million mapped reads (FPKM) within GTEx expression profiles. To compare the expression data from TARGET and GTEx, we executed data merging. We eliminated any probes lacking corresponding gene symbols and averaged the values for genes associated with multiple probes. All the matrix data subsequently underwent a standardization process. This merging and normalization procedure was performed via the limma package of the R program (version 4.0.2; R Core Team, Vienna, Austria; https://www.r-project.org).

### Evaluation of the tumour microenvironment score

Following matrix preprocessing, we assessed stromal and immune scores for osteosarcoma samples from TARGET (TARGET-OS) using the Estimation of STromal and Immune cells in MAlignant Tumor tissues using Expression data (ESTIMATE). ESTIMATE (https://bioinformatics.mdanderson.org/estimate/index.html) is a powerful computational tool designed for resolving the relative proportions of different cell subsets within complex tissues. It provides an accurate quantification of critical aspects within the tumor microenvironment, including tumor purity, as well as the abundance and percentage of stromal and immune cells infiltrating the tumor tissue. This is accomplished through the utilization of deconvolution algorithms on standardized gene expression data [35]. To further investigate and categorize the samples, we categorized them into two separate groups on the basis of the median ESTIMATE scores. We used Student’s t test to analyse the significant differences between the high- and low-score groups. Kaplan-Meier (KM) survival curves were generated to compare survival rates among the high- and low-stromal score groups, high- and low-immune score groups, and overall score groups.

### Identification of differential gene expression

Principal component analysis (PCA) was applied to gain insights into the distinctions between groups in a more compact representation. This technique reduces the dimensionality of the data while retaining critical information. Differential expression analysis was conducted via a classical t test with the limma package in R. We established a significance threshold by requiring |Log2(FoldChange)| > 1 and an adjusted p value (adj. P) < 0.05 for identifying statistically significant gene expression differences. To visually represent the relative expression values and highlight the significant differences, we generated volcano plots and hierarchical clustering heatmaps. These visualizations were created via the ggplot2 and pheatmap packages in R. The intersection or union was visualized via a Venn diagram. To extract tumor microenvironment-related genes (TMIEGs), we merged the differentially expressed genes (DEGs) from the low- and high-stromal and immune groups. The overlapping genes between the TMIEGs and DEGs identified from TARGET-OS and GTEx were regarded as DE-TMIEGs. These DE-TMIEGs represented genes with differential expression patterns that were not only related to the tumor microenvironment but also specific to osteosarcoma.

### Analyses of gene interaction and functional annotation

To investigate the functional interactions among coding-protein genes within the identified set of TMIEGs, we employed the Search Tool for the Retrieval of Interacting Genes (STRING; http://string-db.org). STRING is a web-based platform specifically designed for the analysis of functional associations between genes [12]. The protein‒protein interaction (PPI) network of TMIEGs was established via STRING, enabling a comprehensive understanding of how these genes interact at the molecular level. For a visual representation of these interactions and to integrate them into a more comprehensible framework, we employed Cytoscape, a versatile software tool that serves as an open platform for visualizing biological processes and molecular interaction networks. All the gene nodes were strategically arranged in a circular layout on the basis of their degrees of association, facilitating a clear view of their interactions. To identify densely connected regions and isolate the most significant modules within the network, we utilized the Molecular Complex Detection (MCODE) plugin (version 2.0.2) available in Cytoscape. We set specific criteria for module selection: “MCODE score ≥ 5,” “k score = 2,” “Max depth = 100,” “node score cut-off = 0.2,” and “degree cut-off = 2.” Furthermore, we conducted Gene Ontology (GO) functional enrichment analyses and Kyoto Encyclopedia of Genes and Genomes (KEGG) pathway enrichment analyses for the TMIEGs. These analyses were performed via the Database for Annotation, Visualization, and Integrated Discovery (DAVID; https://david.ncifcrf.gov). DAVID is a valuable web-based resource that helps researchers uncover the genetic significance and functional roles of gene datasets [36]. GO assists in classifying gene biological functions and concepts, making it a useful tool for understanding the roles of the identified genes [37]. On the other hand, KEGG serves as a comprehensive resource for deciphering advanced biological functions and pathways through the analysis of genomic information [38]. The results of these analyses were visually presented via bubble plots, which were generated through the ggplot2 package in R.

### Identification of prognostic TMIELs and TMIEGs

We conducted a screen to identify lncRNAs significantly correlated with the expression levels of DE-TMIEGs in the TARGET-OS dataset on the basis of the Pearson correlation coefficient. The correlation threshold was set to |R| > 0.5 and P < 0.001. The identified lncRNAs were classified as tumor microenvironment-related lncRNAs (TMIELs). Univariate Cox regression analysis was applied in preliminary screening for prognostic TMIELs and TMIEGs. We subsequently applied the Least Absolute Shrinkage and Selection Operator (LASSO) method, which enables optimal factor and feature selection. Through LASSO, we determined the most relevant factors among the many variables under consideration. The number of prognostic gene signatures was established through a 10-fold cross-verification process, leveraging the glmnet package. The final selection of candidate factors closely linked to osteosarcoma prognosis was carried out through multivariate Cox regression analysis. A significance threshold of p < 0.05 was set, ensuring that only the most influential factors were retained. To analyse the correlations between the expression values of prognostic TMIELs and TMIEGs, we utilized Pearson correlation analysis, and the results are presented in an informative heatmap.

### CeRNA networks construction

To explore the interactions between prognostic TMIELs and their potential target microRNAs (miRNAs), we turned to the miRcode resource [37]. miRWalk (http://mirwalk.umm.uni-heidelberg.de) [39], a comprehensive miRNA database that contains target genes for miRNAs from multiple species and provides prediction programs, was used to predict targeted mRNAs positively expressed in miRTarBase, TargetScan, and miRDB [40–42]. Competing endogenous RNAs (ceRNAs) were constructed on the basis of the combination of prognostic TMIELs and targeted miRNAs and mRNAs.

### Assessment and verification of the prognostic signatures

To categorize the samples in the TARGET-OS, we segregated them into high- and low-risk groups using the median risk score as the dividing criterion. PCA was also employed to visually represent the disparities between these groups. Survival disparities between these groups were assessed through the KM curves. A p value < 0.05 was set as the cut-off criterion. A receiver operating characteristic (ROC) curve was generated to evaluate the prediction performance of the prognostic signatures. The Area Under the Curve (AUC) of ROC for the 1-, 3- and 5-year periods was calculated, providing an in-depth analysis of the model’s predictive effectiveness over various timeframes. The associations between survival status and risk score, along with the expression levels of prognostic signatures in the TARGET-OS dataset, are illustrated visually. KM curves were used to show survival probabilities for high- and low-expression prognostic signatures. Univariate and multivariate Cox regression analyses were conducted to determine whether the risk score was an independent prognostic factor while accounting for other clinical factors, such as sex, age, and primary tumor site. Multi-ROC curves of the risk score and clinical factors and the area under the curve (AUC) of each ROC curve for 5 years were generated to present the prediction performance of the risk score. We visually represented the connection between the expression of prognostic signatures and risk scores, alongside clinical variables, via box plots. Finally, we developed a nomogram that integrated prognostic signatures for the prediction of 3- and 5-year survival probabilities in osteosarcoma patients. To ensure the reliability of the model, we employed a calibration curve for verification. The risk score was calculated via the following equation (1) in the R program:


$$Risk\;score\;=\;\sum_{i=1}^{n}\left({Coef}_{i}*x_{i}\right)$$ (1)

the ${Coef}_{i}$ represents the risk coefficient and where $x_{i}$ is the expression value.

An independent dataset (GSE39055) was used to validate the reliability of the identified TMIEGs in predicting OS survival in a different sample population, aiming to reduce the bias that could arise from relying on a single dataset.

### Gene set enrichment analysis and immune infiltration analysis

To unravel the functional disparities between the high- and low-risk groups on the basis of prognostic TMIELs, we harnessed the power of Gene Set Enrichment Analysis (GSEA) software (version 4.3.2) (https://software.broadinstitute.org/gsea/downloads.jsp) to identify critical GO biological processes and KEGG signalling pathways that underlie functional differences. In our analysis, a nominal p value threshold of < 0.05 and a |Normalized Enrichment Score (NES)| > 2 were set as the criteria for selecting the most relevant pathways. To further enhance our understanding of the tumor microenvironment, we turned to Cell-type Identification by Estimating Relative Subsets of RNA Transcripts (CIBERSORT) (http://cibersort.stanford.edu). Launched in 2015, CIBERSORT is a sophisticated computational tool that excels at resolving the proportions of various cell subsets within complex tissues. It accurately predicts the composition of immune cells in the tumor microenvironment and relies on standardized gene expression data and deconvolution algorithms [43]. By analysing the distribution of immune cells via the CIBERSORT algorithm, we gained insights into the cellular landscape within the prognostic TMIELs. In addition to performing a cell composition analysis, we calculated correlations among immune cells via Pearson correlation analysis.

### Ethics approval and consent to participate

This article does not contain any studies with human participants or animals. performed by any of the authors.

## Results

### Data abstraction and preprocessing

We gathered and prepared data, including expression profiles and clinical information, for 88 osteosarcoma samples from the TARGET database (TARGET-OS). As a comparative control group, we obtained RNA sequence data from normal muscle tissues sourced from the GTEx database (GTEx-Muscle), which featured a comprehensive dataset of 396 muscle tissue samples. The TARGET-OS (n = 88) and GTEx-Muscle (n = 396) RNA sequence data were merged.

### Identification of tumor microenvironment-related genes

**Fig 2:**
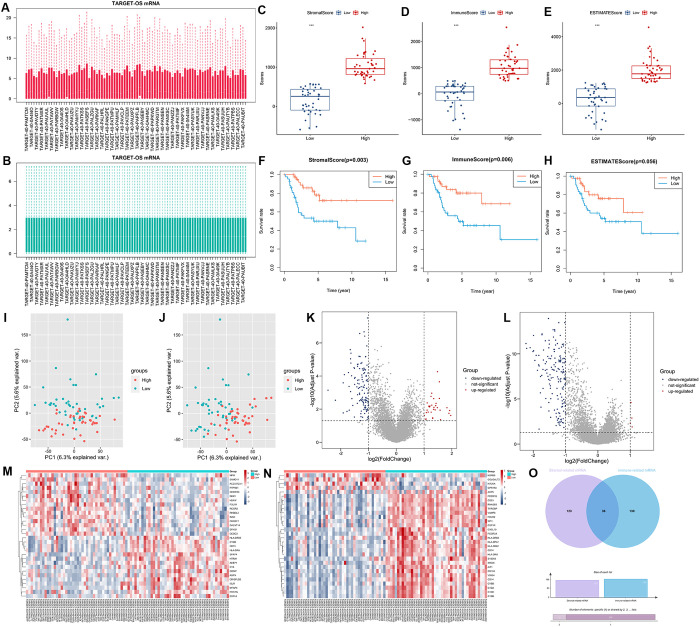
Identification of tumor microenvironment-related genes (TMIEGs). (A, B) Box plots of the expression profiles of OS samples from the TARGET database (TARGET-OS) before normalization and after normalization. (C-E) Box plots of the expression according to the stromal, immune, and ESTIMATE scores between the high- and low-score groups. (F-H) Kaplan–Meier survival curves for the stromal, immune, and ESTIMATE scores between the high- and low-score groups. (I, J) Principal component analyses (PCAs) of the TARGET-OS grouped by the stromal and immune scores. (K, L) Volcano plots of the differentially expressed genes (DEGs) between the high- and low-score groups in both the stromal and immune groups. (M, N) Heatmap of the top 30 DEGs. (O) Venn diagram of TMIEGs.

We presented the expression data of TARGET-OS before and after normalization via box plots (Fig 2A, B). Our analysis of the TARGET-OS dataset via the ESTIMATE tool revealed significant differences (P < 0.001) in the stromal, immune, and ESTIMATE scores between the high- and low-score groups (Fig 2C-E). Survival analysis further revealed that the high-score group experienced the worst outcomes, whether in the context of the immune score, stromal score, or ESTIMATE score groups (Fig 2F-H). PCA revealed distinct distribution patterns between patients with high and low scores in both the stromal and immune groups (Fig 2I, J). We identified 156 DEGs in the stromal groups and 166 DEGs in the immune groups on the basis of the following rigorous criteria: |Log2(FoldChange)| > 1 and an adj. P < 0.05. Among these genes, 124 genes were downregulated, and 32 were upregulated in the stromal groups, whereas 162 genes were downregulated, and 4 were upregulated in the immune groups. These DEGs are visually presented in volcano plots (Fig 2K, L). To provide a focused view, we extracted the expression levels of the top 30 DEGs and presented them with heatmaps (Fig 2M, N). Finally, through a Venn diagram (Fig 2O), we identified a combination of DEGs from the stromal and immune groups, yielding a total of 286 genes. These genes were designated tumor microenvironment-related genes (TMIEGs).

### PPI network construction and functional annotation of TMIEGs

**Fig 3:**
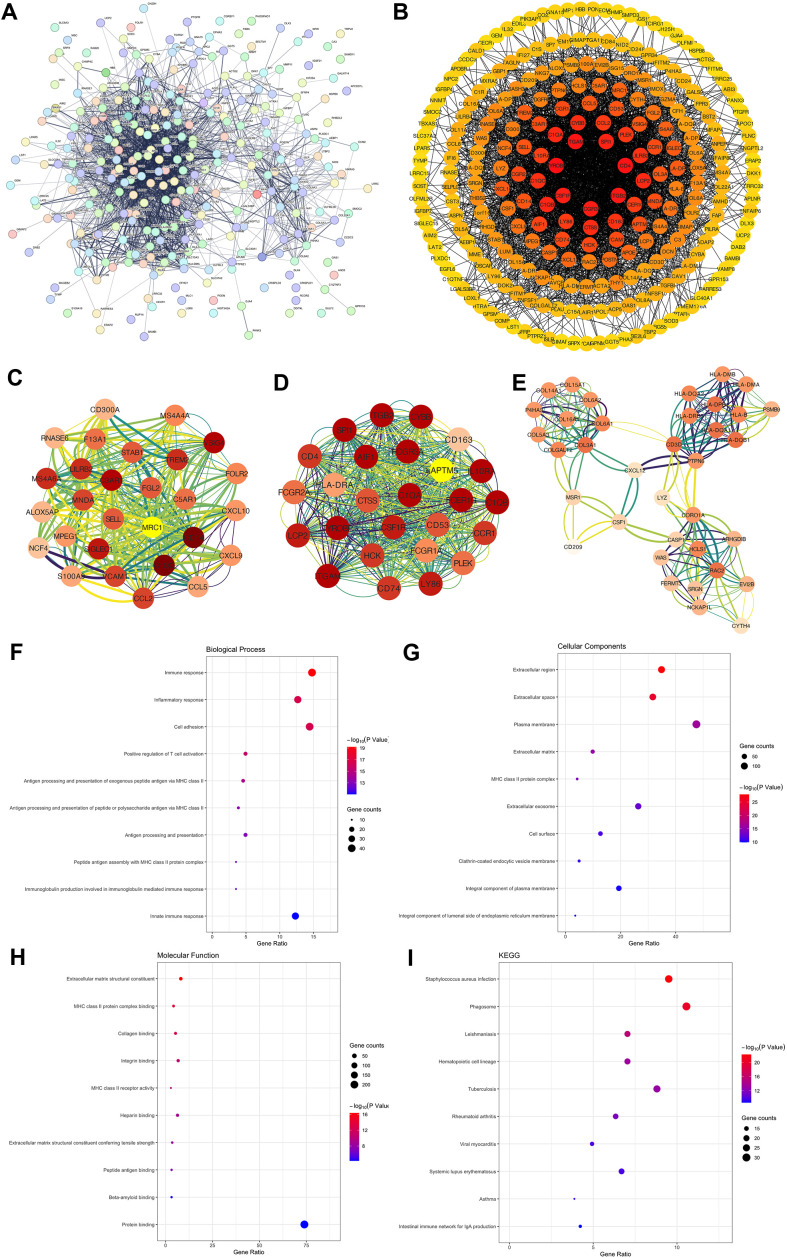
Functional annotation of TMIEGs. (A) Protein‒protein interaction network (PPI) of the TMIEGs. (B) A circular layout of the PPI. (C-E) The top three key functional modules. (F-H) Functional enrichment analyses of the TMIEGs, including biological process, cellular component and molecular function. (I) KEGG pathway analysis.

To unravel the functional significance of the TMIEGs, we constructed a PPI network (Fig 3A). This network was thoughtfully organized into a circular layout on the basis of degrees in Cytoscape (Fig 3B). To pinpoint key functional modules within the PPI network, we extracted the top three modules (Fig 3C-E). We subsequently performed functional and pathway enrichment analyses (Fig 3F-I). In the realm of biological processes (BPs), we observed a strong enrichment of TMIEGs in the immune response (42 genes) and inflammatory response (36 genes), underscoring their roles in the immune system. In terms of cellular components (CCs), TMIEGs were significantly associated with the extracellular region (99 genes) and extracellular space (90 genes), emphasizing their presence and function in the extracellular environment. Within the molecular functions (MFs) category, TMIEGs were notably enriched in extracellular matrix structural constituent (23 genes) and MHC class II protein complex binding (12 genes). Additionally, our KEGG pathway analysis revealed that TMIEGs are involved primarily in pathways associated with immune-mediated diseases. These pathways included staphylococcus aureus infection (27 genes), the phagosome (30 genes), and leishmaniasis (20 genes), highlighting the pivotal role of TMIEGs in immune responses and inflammatory diseases.

### Identification of tumor microenvironment-related lncRNAs

**Fig 4:**
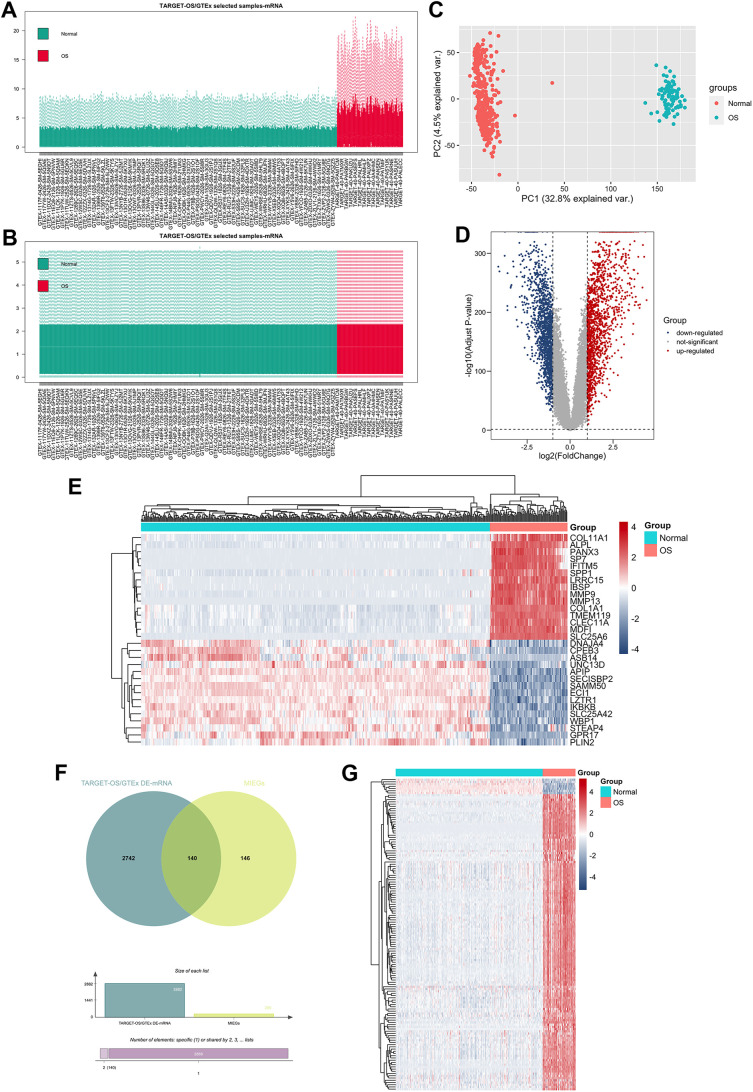
Identification of tumor microenvironment-related lncRNAs (TMIELs). (A, B) Box plots of the osteosarcoma expression profiles from TARGET-OS and normal muscle expression profiles from the Genotype-Tissue Expression database (GTEx) before normalization and after normalization. (C) PCA of the TARGET-OS and GTEx datasets. (D) Volcano plot of the DEGs between TARGET-OS and GTEx. (E) Heatmap of the top 30 DEGs between TARGET-OS and GTEx. (F) Venn diagram of the differentially expressed TMIEGs (DE-TMIEGs). (G) Heatmap of the DE-TMIEGs.

To ensure the quality and comparability of our data, we subjected the merged gene expression data to normalization, as depicted in Figs 4A and B. Following normalization, we employed PCA to illustrate the distribution differences between the two groups (Fig 4C), providing a visual representation of the data’s variance. We identified a total of 2,882 DEGs between these two groups, adhering to the following strict criteria: |Log2(FoldChange)| > 1 and an adj. P < 0.05. These DEGs included 1,422 downregulated genes and 1,460 upregulated genes. We presented the DEGs via a volcano plot and focused on the top 30 DEGs via a heatmap (Fig 4D, E). A Venn diagram of the DEGs and TMIEGs was used to identify the differentially expressed TMIEGs (DE-TMIEGs) (Fig 4F). In total, we obtained 140 DE-TMIEGs, and their expression levels were visually displayed in a heatmap (Fig 4G). We also screened for TMIELs. These genes were selected on the basis of stringent criteria: |R| > 0.5 and P < 0.001, derived from the Pearson correlation coefficient between the expression of DE-TMIEGs and lncRNAs. This comprehensive approach allowed us to identify a total of 380 TMIELs.

### Establishment of prognostic signatures and CeRNA networks

**Fig 5:**
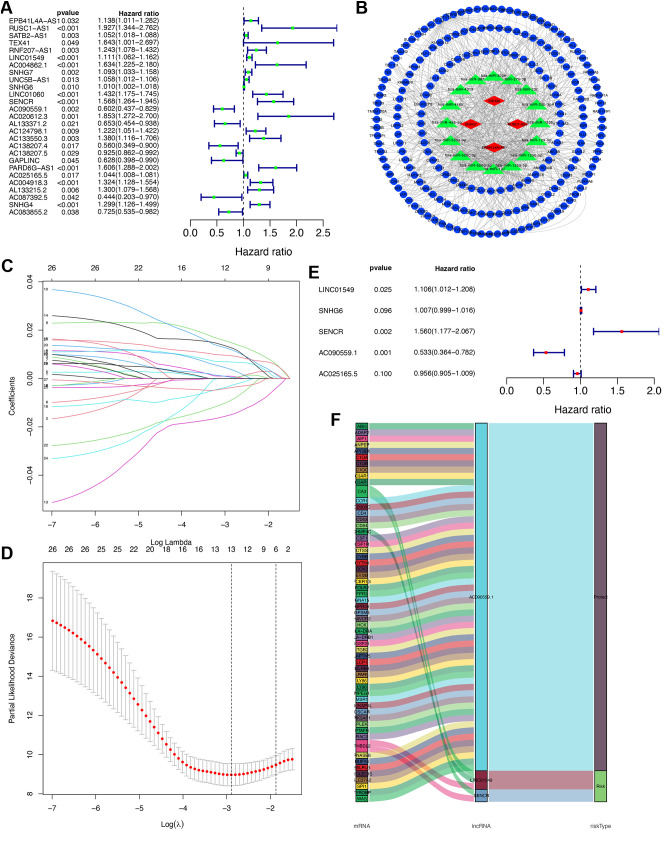
Establishment of prognostic signatures and the ceRNA network. (A) Univariate Cox analysis. (B) A prognostic lncRNA-miRNA-mRNA competing endogenous RNA network (ceRNA). (C, D) Least Absolute Shrinkage and Selection Operator (LASSO) regression for lncRNAs from univariate Cox regression. (E) Multivariate Cox analysis. (F) Sankey plot of prognostic lncRNAs.

We began with a univariate Cox regression analysis of the 380 TMIELs, ultimately resulting in the identification of 27 prognostic TMIELs (Fig 5A). A total of 160 targeted miRNAs were acquired through miRcode on the basis of 27 prognostic lncRNAs (a detailed list is provided in S1 Appendix). We subsequently acquired 198 targeted mRNAs for these miRNAs through the miRWalk database (a detailed list is provided in S2 Appendix), which integrates data from the miRDB, miRTarbase, and TargetScan. This comprehensive approach allowed us to establish a prognostic lncRNA-miRNA-mRNA ceRNA network (Fig 5B). The prognostic lncRNA-miRNA-mRNA ceRNA network may serve as a valuable reference for identifying complex pathways that could influence the prognosis of osteosarcoma patients. Next, we performed LASSO regression analysis, resulting in the further selection of 13 prognostic TMIELs (Fig 5C, D). We subsequently conducted a multivariate Cox analysis on these 13 prognostic TMIELs, ultimately isolating three critical prognostic signatures: AC090559.1, LINC01549, and SENCR (Fig 5E). Hierarchical regression analysis enabled us to effectively refine the crucial lncRNAs potentially involved in the prognosis of osteosarcoma. These three prognostic lncRNAs, along with their coexpressed TMIEGs and their associated risk types, were elegantly visualized via a Sankey plot (Fig 5F). The Sankey plot allowed us to identify how three prognostic lncRNAs regulate specific mRNAs, thereby influencing the prognosis of osteosarcoma, either positively or negatively. AC090559.1 emerged as a protective factor, whereas LINC01549 and SENCR were identified as risk factors.

### Assessment of the prognostic TMIELs

**Fig 6:**
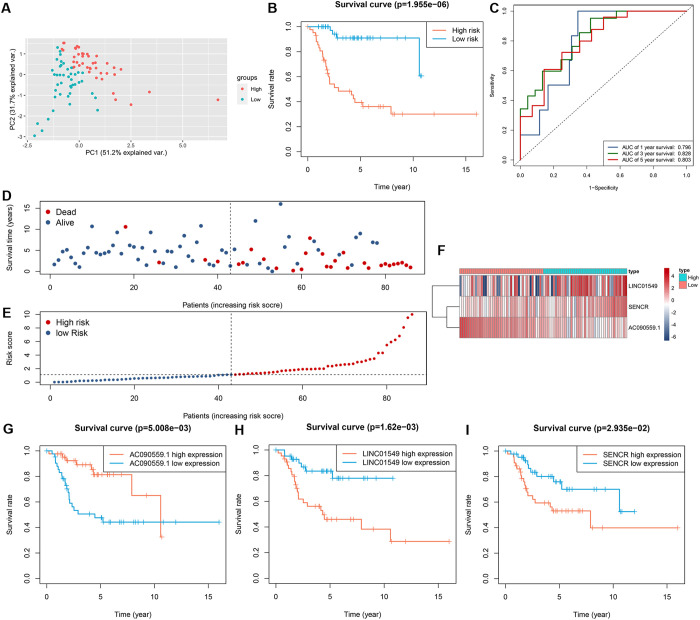
Assessment of the prognostic TMIELs. (A) PCA of the TARGET-OS score grouped by the risk score based on prognostic TMIELs. (B) Kaplan–Meier survival curves of TARGET-OS grouped by risk score on the basis of prognostic TMIELs. (C) The area under the curve (AUC) of the receiver operating characteristic (ROC) curve for the prediction of the 1-, 3- and 5-year survival rates of patients with osteosarcoma. (D, E) Risk score analyses of the prognostic TMIELs. (F) Heatmap of the prognostic TMIELs expressed between the high- and low-risk groups. (G-I) Kaplan–Meier survival curves of three prognostic TMIELs.

PCA clearly revealed distinct expression patterns within the high- and low-risk groups of TARGET-OS patients (Fig 6A). To evaluate the prognostic impact of our identified signatures, we performed a survival analysis. The KM curves strongly depicted a poorer prognosis among high-risk patients (Fig 6B). The prognostic TMIELs exhibited a strong predictive ability for 1-, 3-, and 5-year survival rates among TARGET-OS patients, as clearly illustrated in the ROC curve (Fig 6C). The AUC values for the 1-, 3-, and 5-year survival rates were 0.796, 0.828, and 0.803, respectively. The relationship between survival status and risk score is shown in the dot plot (Fig 6D-E). For a detailed look at the expression of the three critical prognostic TMIELs, we utilized a heatmap (Fig 6F). The KM curves demonstrated that AC090559.1, which acts as a protective factor, was significantly associated with improved survival in osteosarcoma patients. In contrast, LINC01549 and SENCR, identified as risk factors, were significantly negatively correlated with patient survival (Fig 6G-I).

### Clinical relevance of prognostic TMIELs

**Fig 7:**
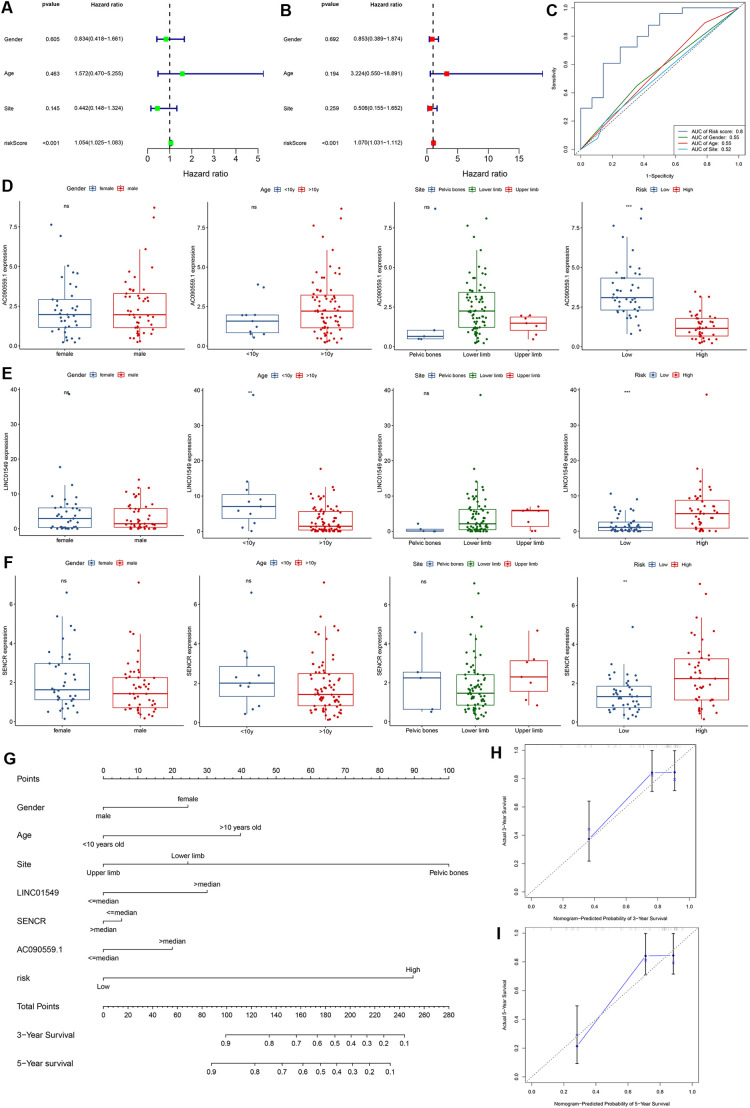
Clinical evaluation of the prognostic TMIELs. (A, B) Univariate and multivariate Cox analyses of the prognostic TMIELs and clinical characteristics. (C) AUC of the ROC curve for the prediction of the 1-, 3- and 5-year survival rates of patients with osteosarcoma on the basis of the prognostic TMIEL score and clinical characteristics. (D-F) The expression levels of the three prognostic TMIELs between groups were grouped according to sex, age (< 10 y; ≥ 10 y), sex, site, and risk. (G) Nomogram according to sex, age, site, and risk. (H, I) Calibration plots of the nomogram for predicting the probability of survival at 3 and 5 years.

Our analysis revealed that the risk score of the prognostic TMIELs was an independent factor, which was validated through both univariate and multivariate Cox regression analyses (Fig 7A, B). By comparing the predictive capacity of our prognostic TMIELs with that of clinical signatures, we found that the TMIELs outperformed the clinical factors (Fig 7C). The boxplots revealed that AC090559.1 was expressed at significantly higher levels in low-risk patients (P < 0.001), whereas LINC01549 and SENCR were notably upregulated in high-risk patients (P < 0.01) (Fig 7D-F). Furthermore, our analysis revealed that the expression of these prognostic TMIELs was largely independent of clinical characteristics, except for LINC01549, which was significantly more highly expressed in patients younger than 10 years (P < 0.01). These findings emphasize the potential age-related impact on LINC01549 expression. We constructed a nomogram model for the prediction of 3- and 5-year survival in osteosarcoma patients via multivariate Cox regression (Fig 7G). The predictive factors incorporated into this model included sex, age, the primary site of osteosarcoma, the expression levels of the three prognostic TMIELs, and the risk level. The high prediction accuracy of the nomogram was validated through well-calibrated curves (Fig 7H, I).

### Gene set enrichment analysis and immune infiltration analysis

**Fig 8:**
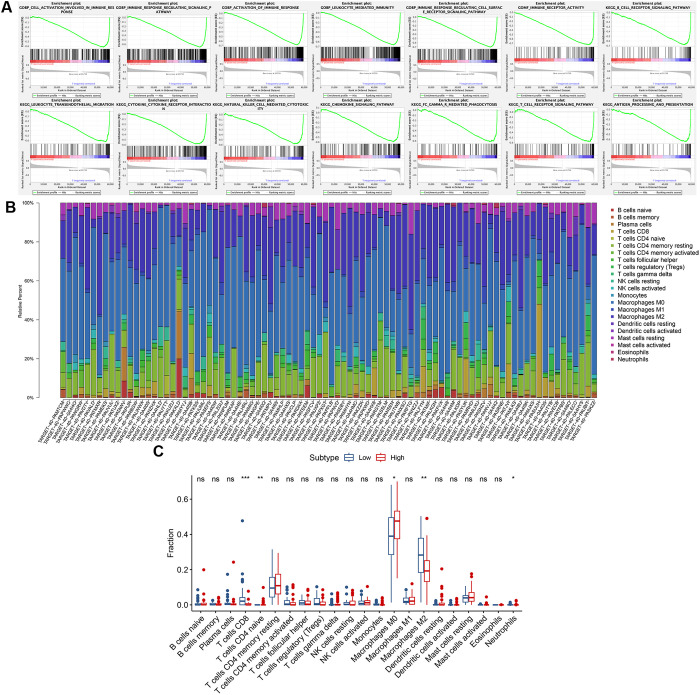
Gene set enrichment analysis (GSEA) and immune infiltration analysis based on the prognostic TMIELs. (A) GSEA results. (B) The distribution of immune cells. (C) Box plots of the relationships between risk type and immune-related cells.

GSEA revealed functional disparities between the low- and high-risk groups (Fig 8A). In the low-risk group, BPs were predominantly involved in immune-related responses and pathways, encompassing cell activation involved in the immune response, immune response-regulating signalling pathways, activation of the immune response, leukocyte-mediated immunity, and immune response-regulating cell surface receptor signalling pathways. The low-risk group also exhibited notable enrichment in immune receptor activity within the MFs. Furthermore, the KEGG pathways in the low-risk group were significantly enriched in immune cells and factor-related processes. These pathways included the B-cell receptor signalling pathway, leukocyte transendothelial migration, cytokine‒cytokine receptor interaction, natural killer cell-mediated cytotoxicity, the chemokine signalling pathway, Fcγ receptor-mediated phagocytosis, the T-cell receptor signalling pathway, and antigen processing and presentation. These findings suggest that the activation of immune regulatory processes can mitigate the risk for osteosarcoma patients and improve their prognosis. To further explore the immune landscape, we employed the CIBERSORT algorithm to assess the distribution of immune cells (Fig 8B). Macrophages and T cells represented the major components. We further examined the associations between risk type and immune-related cells through boxplots (Fig 8C). Significant differences in CD8 + T cells, naive CD4 + T cells, M0 macrophages, M2 macrophages, and neutrophils were detected between the low- and high-risk groups.

### Identification of prognostic TMIEGs

**Fig 9:**
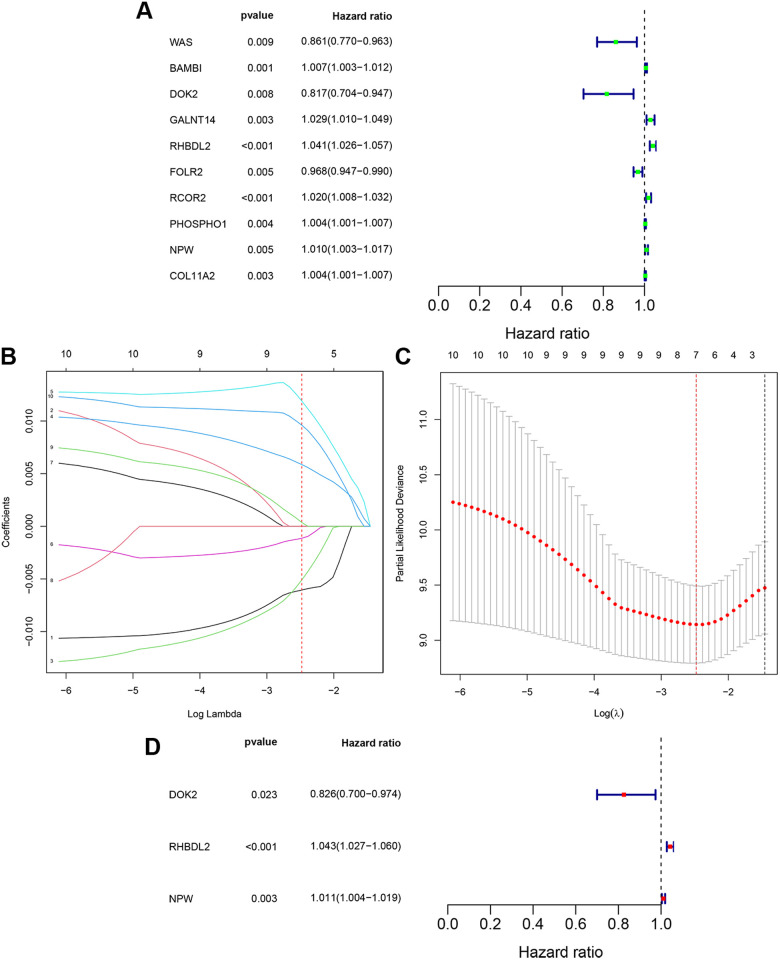
Identification of prognostic TMIEGs. (A) Univariate Cox analysis. (B, C) LASSO regression for TMIEGs from univariate Cox regression. (D) Multivariate Cox analysis.

Univariate Cox regression analysis of the 140 DE-TMIEGs was performed, and 10 potential prognostic TMIEGs were obtained (Fig 9A). We then utilized LASSO regression analysis to refine the selection. This process led to the identification of 7 robust prognostic TMIEGs (Fig 9B, C), narrowing the list to genes with high prognostic relevance. To arrive at a definitive prognostic model, multivariate Cox analysis was employed, culminating in the identification of three crucial prognostic signatures: DOK2, RHBDL2, and NPW (Fig 9D).

### Assessment of prognostic TMIEGs

**Fig 10:**
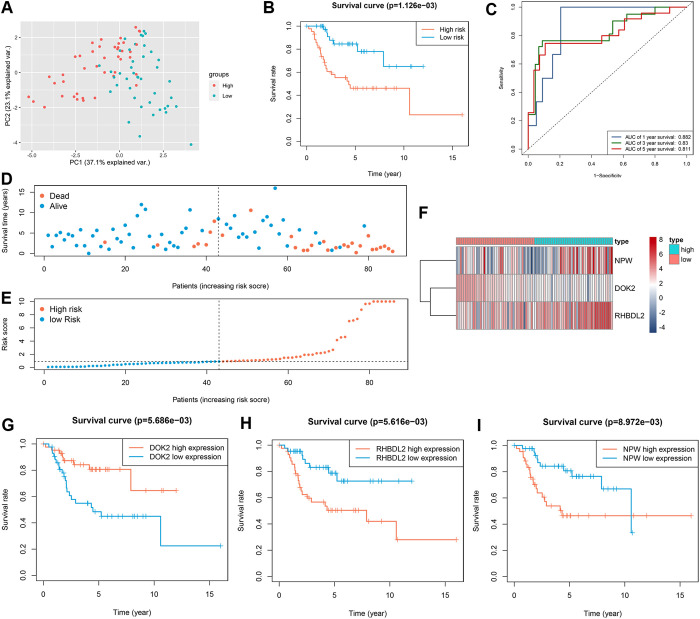
Assessment of prognostic TMIEGs. (A) PCA of the TARGET-OS grouped by the risk scores based on prognostic TMIEGs. (B) Kaplan–Meier survival curves of TARGET-OS grouped by risk score on the basis of prognostic TMIEGs. (C) AUC of the ROC curve for the prediction of the 1-, 3- and 5-year survival rates of patients with osteosarcoma. (D, E) Risk score analyses of the prognostic TMIEGs. (F) Heatmap of the prognostic TMIEGs expressed between the high- and low-risk groups. (G-I) Kaplan–Meier survival curves of three prognostic TMIEGs.

PCA was employed to visualize the distinctive expression profiles within the high- and low-risk groups of TARGET-OS patients (Fig 10A). The risk grouping was predicated on the prognostic TMIEGs. The KM curves of survival analysis unequivocally highlighted a poorer prognosis among high-risk patients (Fig 10B), underscoring the clinical significance of the prognostic TMIEGs. The prognostic TMIEGs demonstrated robust predictive capabilities for 1-, 3-, and 5-year survival rates among TARGET-OS patients, as evident in the ROC curve (Fig 10C). The AUC values for the 1-, 3-, and 5-year survival rates were 0.882, 0.830, and 0.811, respectively, confirming the accuracy and reliability of the model. The relationship between survival status and risk score was effectively visualized in a dot plot (Fig 10D-E). A heatmap was generated to display the expression levels of the three pivotal prognostic TMIEGs (Fig 10F). The KM curves revealed that DOK2, which is a protective factor, was significantly positively correlated with OS survival, whereas RHBDL2 and NPW, which are risk factors, were notably negatively correlated with patient survival (Fig 10G-I).

### Clinical relevance of prognostic TMIEGs

**Fig 11:**
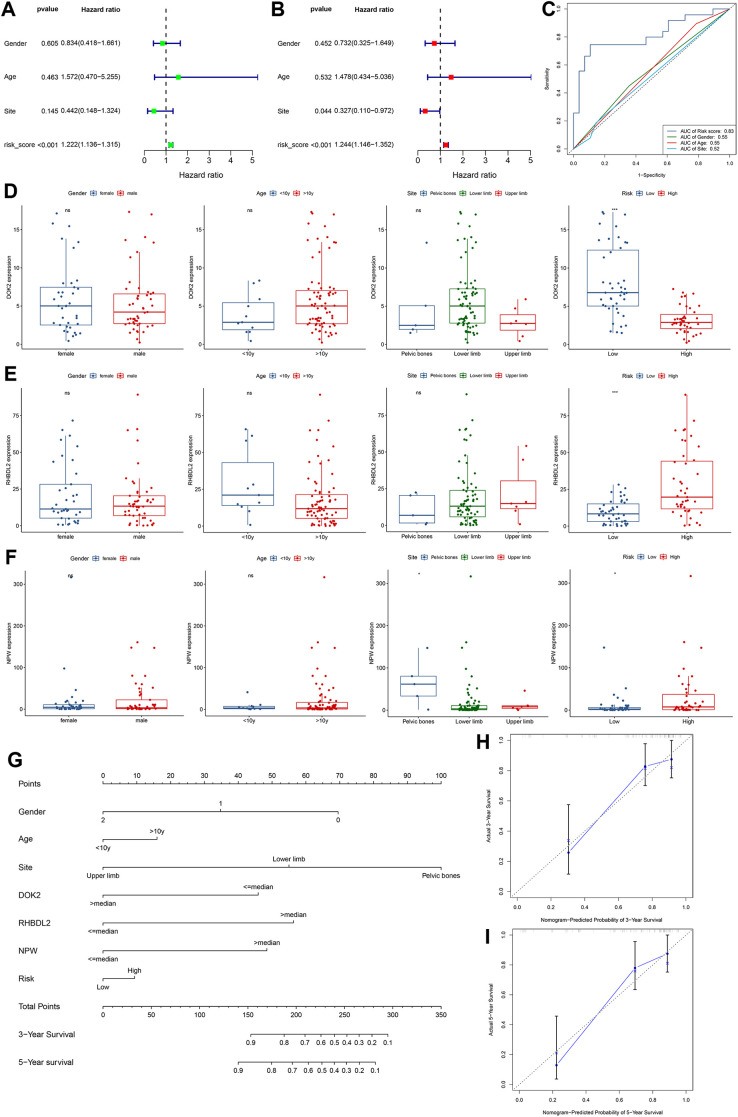
Clinical evaluation of the prognostic TMIEGs. (A, B) Univariate and multivariate Cox analyses of the prognostic TMIEGs and clinical characteristics. (C) AUC of the ROC curve for the prediction of the 1-, 3- and 5-year survival rates of patients with osteosarcoma on the basis of the prognostic TMIEGs and clinical characteristics. (D-F) The expression levels of the three prognostic TMIEGs between groups were grouped according to sex, age (< 10 y; ≥ 10 y), sex, site, and risk. (G) Nomogram according to sex, age, site, and risk. (H, I) Calibration plots of the nomogram for predicting the probability of survival at 3 and 5 years.

As shown by univariate and multivariate Cox regression analyses (Fig 11A, B), the risk score of the prognostic TMIEGs was an excellent predictor. Additionally, the primary site of the tumor emerged as a determinant of prognosis, notably influencing patient outcomes, as identified in the multivariate analysis. When the predictive capacity of the prognostic TMIEGs was compared with that of the clinical signatures, we found that the TMIEGs outperformed the clinical factors, further emphasizing the superiority of the molecular signatures in predicting patient prognosis (Fig 11C). The boxplots effectively revealed that DOK2 was significantly upregulated in low-risk patients (P < 0.001), whereas RHBDL2 and NPW were notably upregulated in high-risk patients (P < 0.05) (Fig 11D-F). The expression of these prognostic TMIEGs was largely independent of clinical characteristics, except for the NPW, which exhibited significantly greater expression in patients with pelvic tumors at the primary site (P < 0.05). This insight highlights the potential role of the NPW in the context of primary pelvic tumors. On the basis of multivariate Cox regression analysis, we constructed a nomogram model for the prediction of 3- and 5-year survival in osteosarcoma patients (Fig 11G). The model incorporated predictive factors such as sex, age, primary tumor site, the expression levels of the three crucial prognostic TMIEGs, and the risk level. Notably, the high prediction accuracy of the nomogram was confirmed through well-calibrated curves (Fig 11H, I).

### Correlation between prognostic TMIELs and TMIEGs

**Fig 12:**
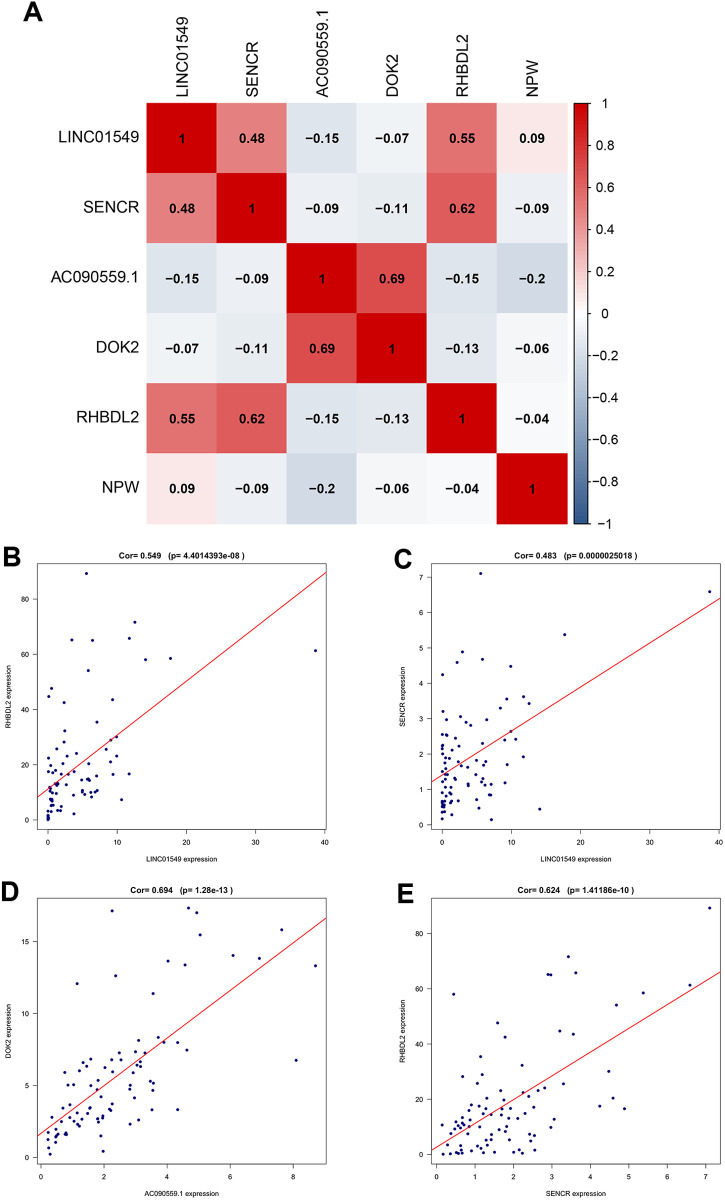
Correlation analysis of prognostic TMIELs and TMIEGs. (A) Pearson correlation coefficients between the prognostic TMIELs and TMIEGs. (B-E) Correlation of the expression between the prognostic TMIELs and TMIEGs.

Pearson correlation analysis was employed to elucidate these associations between the expression of prognostic TMIELs and TMIEGs, as shown in Fig 12A. The expression of the lncRNA LINC01549 was positively correlated with the expression of the RHBDL2 mRNA and the lncRNA SENCR. Similarly, the expression of the lncRNA AC090559.1 was positively correlated with the expression of the DOK2 mRNA. Moreover, the lncRNA SENCR was positively associated with the expression of the RHBDL2 mRNA. These findings are visually represented in Fig 12B-E.

### Validation of the prognostic TMIEGs

**Fig 13:**
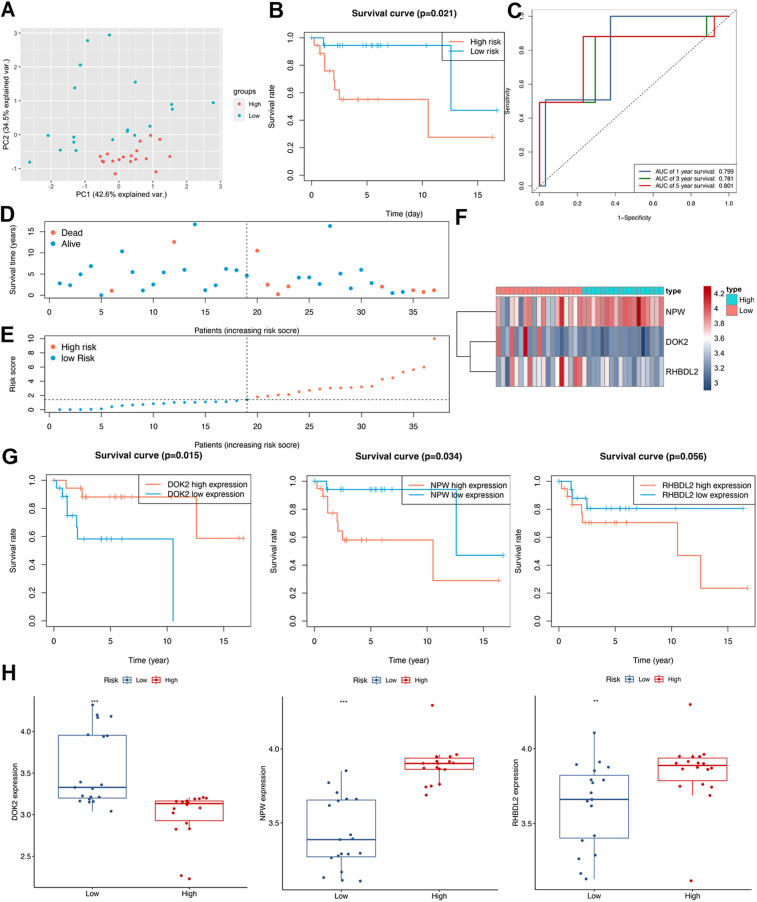
Validation of prognostic TMIEGs. (A) PCA of GSE39055 grouped by risk score on the basis of prognostic TMIEGs. (B) Kaplan–Meier survival curves of GSE39055 patients grouped by risk score on the basis of prognostic TMIEGs. (C) AUC of the ROC curve for the prediction of the 1-, 3- and 5-year survival rates of patients with osteosarcoma. (D, E) Risk score analyses of the prognostic TMIEGs. (F) Heatmap of the prognostic TMIEGs expressed between the high- and low-risk groups. (G) Kaplan–Meier survival curves of three prognostic TMIEGs. (H) The expression levels of the three prognostic TMIEGs between groups were grouped according to risk.

An independent dataset (GSE39055) was used to validate the reliability of the identified TMIEGs in predicting osteosarcoma prognosis. According to the expression levels of three prognostic TMIEGs (DOK2, NPW and RHBDL2) in GSE39055, the risk score was calculated for each sample via equation (1) in the R program. PCA clearly revealed distinct expression patterns between the high- and low-risk groups among the GSE39055 patients (Fig 13A). The KM curves clearly demonstrated a significantly poorer prognosis for high-risk patients (Fig 13B). There was a strong ability of the prognostic TMIEGs to predict 1-, 3-, and 5-year survival rates in GSE39055 patients, as clearly shown by the ROC curve (Fig 13C). The AUC values for the 1-, 3-, and 5-year survival rates were 0.799, 0.781, and 0.801, respectively. The relationship between survival status and risk score is illustrated in the dot plot (Fig 13D-E). To provide a detailed view of the expression of the three critical prognostic TMIEGs, a heatmap was generated (Fig 13F). The KM curve indicated that DOK2 acted as a protective factor, whereas NPW and RHBDL2 functioned as risk factors, all of which were associated with the survival rate of osteosarcoma patients. Both DOK2 and NPW were statistically significant (P < 0.05), while the p value for RHBDL2 was 0.054 (Fig 13G). In the GSE39055 dataset, box plots also effectively indicated that DOK2 was significantly upregulated in low-risk patients (P < 0.001), whereas RHBDL2 and NPW were significantly upregulated in high-risk patients (P < 0.05 and P < 0.001, respectively). (Fig 13H).

## Discussion

Osteosarcoma, recognized as the primary malignant bone tumor, continues to pose a significant threat, with high mortality rates among children and adolescents [44]. The overall prognosis for patients is generally bleak, with a 5-year survival rate of approximately 50%. This grim outlook is largely attributed to the aggressive metastatic and highly invasive characteristics of osteosarcoma [45–47]. Regrettably, there have been no notable breakthroughs in the treatment of osteosarcoma in recent years, highlighting an urgent imperative to develop innovative and effective strategies that can address the current clinical challenges. The identification of novel molecular biomarkers holds substantial promise for advancing the diagnosis, treatment, and assessment of metastasis in osteosarcoma. Notably, the tumor microenvironment is crucial in the progression of osteosarcoma and may be strongly associated with the development of drug resistance during treatment [48]. However, a glaring gap exists in our current understanding, as there are no confirmed tumor microenvironment-related genes or lncRNAs, nor have their interactions been delineated in the context of osteosarcoma. Furthermore, few studies have explored the correlation between these molecular entities and the overall prognosis of osteosarcoma patients, especially those based on comprehensive datasets such as those available in the TARGET and GTEx databases. Addressing these knowledge gaps holds great promise for advancing our understanding and ultimately improving the management of osteosarcoma.

In this study, we harnessed the combined power of RNA sequence data from the TARGET-OS cohort (comprising 88 samples) and the GTEx-Muscle dataset (comprising 396 samples) to perform a comprehensive analysis. The application of ESTIMATE analysis to TARGET-OS and subsequent survival analysis revealed a significant association between the survival duration of osteosarcoma patients and the ESTIMATE score, reaffirming the pivotal role of the tumor microenvironment in determining the prognosis of osteosarcoma patients. Following this validation, we embarked on a systematic refinement of a comprehensive set of 286 DEGs specifically related to the osteosarcoma TME. These DEGs were notably enriched in immune- and stromal-mediated processes, as revealed through functional annotation. Further amalgamation of the data from TARGET-OS and GTEx allowed us to identify 140 DE-TMIEGs that were differentially expressed between the two datasets. Moreover, we identified 380 lncRNAs associated with these DE-TMIEGs. The subsequent application of univariate Cox regression analysis yielded a list of 27 prognostic TMIELs. To delve deeper into these regulatory networks, we identified the miRNAs and mRNAs targeted by these prognostic TMIELs, facilitating the construction of a ceRNA network. Notably, our ceRNA network was relatively incomplete, consisting of only 4 lncRNAs, 16 miRNAs, and 198 mRNAs, and even inconsistent with the coexpressed TMIEGs. This was potentially due to the limitations imposed by existing databases, which may not capture the full spectrum of interactions in the complex osteosarcoma microenvironment. In the pursuit of identifying robust prognostic markers, LASSO regression analysis and multivariate Cox analysis sequentially indicated that 3 TMIELs, AC090559.1, LINC01549 and SENCR, were good candidates for prognostic markers. In assessing variables affecting patient prognosis, we typically start with univariate Cox analysis to pinpoint significant associations with survival. A multivariate model was subsequently constructed to validate the independence of these variables concerning survival. However, when dealing with multicollinearity or a high number of variables compared with the sample size, we employed LASSO regression. LASSO can be used to screen variables effectively while considering multicollinearity, and then a multivariate Cox regression model was used for analysis, enhancing our ability to identify significant prognostic factors. This methodological refinement bolsters the reliability and accuracy of our findings. Unfortunately, we do not have sufficient data with survival characteristics to validate the identified prognostic TMIELs, either from public databases or our own experimental data.

AC090559.1 emerged as a protective factor, whereas LINC01549 and SENCR were identified as risk factors and exhibited strong predictive potential. According to the three prognostic TMIEL models, high-risk patients had notably poorer outcomes than low-risk patients did. These prognostic signatures were independent of other clinical characteristics. GSEA revealed that immune process activation plays a pivotal role in mitigating the risk associated with osteosarcoma, leading to improved patient prognosis. Furthermore, the distribution of immune cells was explored. Macrophages and T cells were the predominant cell types. Low-risk patients presented significantly higher levels of CD8 + T cells and macrophages M2, whereas CD4 + T cells, macrophages M0, and neutrophils were significantly lower in the low-risk group than in the high-risk group, indicating that systemic immune dysfunction in high-risk patients could impact their prognosis. In conclusion, the three prognostic TMIELs present a robust means to accurately predict overall survival in patients with osteosarcoma.

Notably, AC090559.1 has featured prominently in recent studies, indicating its importance in various aspects of tumor biology. Wu *et al*. [49] developed an autophagy-related lncRNA signature featuring AC090559.1, which has potential for predicting the prognosis of lung adenocarcinoma. Additionally, AC090559.1 was identified as one of the 10 prognostic ferroptosis-related lncRNAs in the tumor microenvironment of lung adenocarcinoma [50]. Furthermore, AC090559.1 has emerged as a predictive factor for prognosis and is linked to pyroptosis and disulfidptosis in lung adenocarcinoma [51,52]. In the context of osteosarcoma, AC090559.1 was identified as a novel necroptosis-related lncRNA signature [53]. These collective findings suggest the pivotal role of AC090559.1, an essential lncRNA, in influencing tumor progression through its intricate interactions within the TME. SENCR, which has been shown to significantly influence vascular smooth muscle cell (VSMC) phenotypes and vascular remodelling [54], is termed “Angio-LncRNA” and plays an important role in vascular diseases, such as diabetic cardiomyopathy, atherosclerotic coronary artery disease and Jacobsen syndrome [55–60]. SENCR, initially identified in 2014, is a cytoplasmic lncRNA enriched in vascular cells that contributes to stabilizing the smooth muscle cell contractile phenotype [61]. Notably, its downregulation has been associated with enhanced smooth muscle cell proliferation and migration, as evidenced in db/db mice through the upregulation of FoxO1 and TRPC6 [62]. SENCR also plays a key role in regulating endothelial differentiation from pluripotent cells and controlling the angiogenic capacity of human umbilical endothelial cells [63]. In the context of cancer, SENCR has been implicated in promoting the proliferation and progression of non-small cell lung cancer (NSCLC) cells through its interaction with miR-1-3p [64]. It was found to be upregulated in A549/DDP cells, contributing to cisplatin resistance and the growth of NSCLC cells by upregulating FLI1 expression [65]. Furthermore, SENCR and genetic variants of IGF2 BP3 were identified as potential risk factors for Ewing sarcoma development and disease progression, highlighting the role of heritable factors in influencing susceptibility to Ewing sarcoma and predicting patient prognosis [66]. Additionally, SENCR was also identified as part of a necroptosis-related lncRNA signature, similar to AC090559.1 [53]. However, LINC01549 has been less explored in the literature. A risk scoring system that incorporates LINC01549 has demonstrated its effectiveness in predicting overall survival in hepatocellular carcinoma patients with cirrhosis [67].

In this study, we identified 286 TMEIGs and 140 DE-TMIEGs. Functional enrichment analysis of the TMIEGs revealed enriched GO terms and signalling pathways associated with immunity and inflammation. Through a series of analyses, we identified prognostic markers composed of three TMIEGs: DOK2, RHBDL2, and NPW. These signatures yielded risk scores, categorizing patients into high-risk and low-risk groups. Survival analysis clearly demonstrated that high-risk patients had a more unfavourable prognosis. Among the three TMIEGs, DOK2 acted as a protective factor, whereas RHBDL2 and NPW were identified as risk factors. Univariate Cox regression analysis indicated that our signatures were independent prognostic factors compared with other clinical signatures. In addition, multivariate Cox regression analysis suggested that our signatures and the primary tumor site were two independent prognostic factors. Three prognostic TMIEGs were validated through an independent dataset.

DOK2, a member of the downstream tyrosine kinase family, is involved in various physiological functions, with a particular focus on its role in negatively regulating the T-cell signalling pathway and influencing the growth and development of hematopoietic progenitor cells [68]. DOK2 has been implicated in a spectrum of diseases. Research has revealed that DOK2 is a target of copy number loss and mRNA downregulation, effectively suppressing the proliferation of lung cancer cells both in vitro and in vivo [69,70]. The overexpression of region-specific DOK2 has been linked to poor prognosis in human astrocytoma [71] and has been recognized as a prognostic gene within the tumor microenvironment of lung adenocarcinomas [72]. The involvement of DOK2 in chemotherapy resistance extends across various diseases. Its loss can induce resistance to chemotherapy by reducing apoptosis levels in response to treatment [73]. Conversely, in ovarian cancer, the upregulation of DOK2 sensitizes ovarian cancer cells to platinum-based drugs, potentially increasing the effectiveness of treatment [74]. In other contexts, DOK2 may serve as a valuable prognostic marker for patients undergoing curative resection for conditions such as colorectal cancer and gastric adenocarcinoma [75,76]. Moreover, the combined negative expression of DOK2 and RASA1 may function as an independent prognostic factor for patients after breast cancer surgery [77]. In summary, DOK2 holds considerable promise as a reference for assessing patient prognosis in surgical settings. RHBDL2, a member of the rhomboid protein family, plays a multifaceted role in keratinocyte proliferation, and its involvement in cancer has been documented [78]. Notably, RHBDL2 also functions as a critical component of cancer-associated fibroblasts, a prominent element within the tumor microenvironment with versatile roles in tumor progression [79]. In various tumor cell lines, endogenous RHBDL2 activity has been observed, where it cleaves EGF just outside its transmembrane domain, promoting its secretion and triggering EGFR activation [80]. Furthermore, RHBDL2 plays a crucial role in pancreatic cancer, where it enhances proliferation, migration, and invasion by stabilizing N1ICD through interactions with OTUD7B and activation of the Notch signalling pathway [81]. NPW, a novel endogenous peptide known to interact with the G protein-coupled receptors GPR7 (NPBWR1) and GPR8 (NPBWR2), has been identified in the gastric antral mucosa of the stomach in rats, mice, and humans [82]. This neuropeptide system is believed to play a role in the regulation of feeding behavior, maintenance of energy homeostasis, neuroendocrine functions, and modulation of inflammatory pain [83]. The NPW, NPB, and NPBWR1 systems are directly associated with the regulation of proliferative activity in cultured rat calvaria osteoblast-like cells [84]. In the context of cancer, NPW has been found to be coexpressed in ovarian cancer stem cells, suggesting its potential as a drug target for ovarian cancer [85]. However, the effects of the NPW on osteosarcoma remain unexplored, necessitating further studies to comprehensively investigate its impact in this specific context.

Currently, several prognostic models have been developed for osteosarcoma. These include a model comprising 13 immune-related genes [86], as well as predictive models based on autophagy-related genes, lncRNAs [87], and anoikis-related lncRNAs [88]. However, a tumor microenvironment-related prognostic lncRNA model has not been previously established. The expression of the lncRNA LINC01549 was positively correlated with the expression of the RHBDL2 mRNA and the lncRNA SENCR. Furthermore, the expression of the lncRNA AC090559.1 was positively associated with the expression of the DOK2 mRNA, and the expression of the lncRNA SENCR was positively correlated with the expression of the RHBDL2 mRNA. However, notably, there is currently a dearth of research elucidating the specific interactions and networks involving these components. Further studies are warranted to explore these intricate relationships.

In this study, for the first time, we combined the TARGET tumor dataset and the GTEx normal tissue dataset to create a prognostic model for tumor microenvironment-related markers in osteosarcoma. While the study successfully developed this model, there are a few limitations to consider. First, the disparity in sample size between healthy (n = 396) and osteosarcoma (n = 88) tissue samples in the GTEx and TARGET databases could impact the results. Future analyses with a larger number of tumor samples are necessary to increase the robustness of the model. Second, the absence of lncRNA expression data in external datasets limits the verification of tumor microenvironment-related lncRNA models, necessitating the verification of more lncRNA expression profiles in osteosarcoma.

## Conclusion

Our study offers new insights into the complex landscape of osteosarcoma prognosis, unveiling a comprehensive set of TMIEGs and their associated TMIELs as potent predictors of patient survival. Our three-member prognostic TMIEL panel, AC090559.1, LINC01549, and SENCR, exhibit robust prognostic accuracy in distinguishing high-risk and low-risk osteosarcoma patients. Three prognostic TMIEGs, DOK2, RHBDL2, and NPW, also perform similarly. We have also highlighted the pivotal role of immune processes in osteosarcoma and the intriguing distribution of immune cells. This work not only offers valuable prognostic markers but also sets the stage for further investigations into the intricate relationships between these molecules. Ultimately, these findings underscore the pivotal role of the tumor microenvironment in osteosarcoma and underscore the potential utility of TMIEGs and TMIELs in osteosarcoma therapy.

## Supporting information

S1 Appendix The detailed list of 160 targeted miRNAs. (DOCX)

S2 Appendix The detailed list of 198 targeted mRNAs. (DOCX)
