# Expression of Concern: Signatures of tumor microenvironment-related genes and long noncoding RNAs predict poor prognosis in osteosarcoma

**DOI:** 10.1371/journal.pone.0354638

**Published:** 2026-07-27

**Authors:** The PLOS One Editors

After this article [1] was published, the following concerns were noted:

- The articles cited as References 53 and 64 were retracted before [1] was published.
- The article cited as Reference 29 was retracted after [1] was published.

In addition, the *PLOS One* Editors have concerns about the article’s peer review. The Editors do not have concerns regarding the authors’ conduct during the peer review process.

The third author stated that the three retracted articles cited in [1] were cited for general background introduction.

In light of the cumulative issues, the *PLOS One* Editors issue this Expression of Concern. Readers are advised to interpret the article [1] with caution.
