# Supplementary material for: Signatures of tumor microenvironment-related genes and long noncoding RNAs predict poor prognosis in osteosarcoma
Source: PLoS One. 2025 Jul 16;20(7):e0326876. doi: 10.1371/journal.pone.0326876 (PMC12266395; doi:10.1371/journal.pone.0326876)
Supplement: S1 Appendix — (DOCX) [file pone.0326876.s001.docx]

hsa-miR-503

hsa-miR-7

hsa-miR-7ab

hsa-miR-9

hsa-miR-9ab

hsa-miR-93

hsa-miR-93a

hsa-miR-105

hsa-miR-106a

hsa-miR-291a-3p

hsa-miR-294

hsa-miR-295

hsa-miR-302abcde

hsa-miR-372

hsa-miR-373

hsa-miR-428

hsa-miR-519a

hsa-miR-520be

hsa-miR-520acd-3p

hsa-miR-1378

hsa-miR-1420ac

hsa-miR-139-5p

hsa-miR-141

hsa-miR-200a

hsa-miR-146ac

hsa-miR-146b-5p

hsa-miR-148ab-3p

hsa-miR-152

hsa-miR-150

hsa-miR-5127

hsa-miR-15abc

hsa-miR-16

hsa-miR-16abc

hsa-miR-195

hsa-miR-322

hsa-miR-424

hsa-miR-497

hsa-miR-1907

hsa-miR-17

hsa-miR-17-5p

hsa-miR-20ab

hsa-miR-20b-5p

hsa-miR-106ab

hsa-miR-427

hsa-miR-518a-3p

hsa-miR-519d

hsa-miR-183

hsa-miR-194

hsa-miR-19ab

hsa-miR-200bc

hsa-miR-429

hsa-miR-548a

hsa-miR-205

hsa-miR-205ab

hsa-miR-208ab

hsa-miR-208ab-3p

hsa-miR-214

hsa-miR-761

hsa-miR-3619-5p

hsa-miR-217

hsa-miR-218

hsa-miR-218a

hsa-miR-22

hsa-miR-22-3p

hsa-miR-223

hsa-miR-23abc

hsa-miR-23b-3p

hsa-miR-30abcdef

hsa-miR-30abe-5p

hsa-miR-384-5p

hsa-miR-338

hsa-miR-338-3p

hsa-miR-34ac

hsa-miR-34bc-5p

hsa-miR-449abc

hsa-miR-449c-5p

hsa-miR-383

hsa-miR-425

hsa-miR-425-5p

hsa-miR-489

hsa-miR-129-5p

hsa-miR-129ab-5p

hsa-miR-499-5p

hsa-miR-96

hsa-miR-507

hsa-miR-1271

hsa-miR-135ab

hsa-miR-135a-5p

hsa-miR-138

hsa-miR-138ab

hsa-miR-155

hsa-miR-181abcd

hsa-miR-4262

hsa-miR-182

hsa-miR-18ab

hsa-miR-4735-3p

hsa-miR-192

hsa-miR-215

hsa-miR-193

hsa-miR-193b

hsa-miR-193a-3p

hsa-miR-199ab-5p

hsa-miR-203

hsa-miR-216a

hsa-miR-216b

hsa-miR-216b-5p

hsa-miR-25

hsa-miR-32

hsa-miR-92abc

hsa-miR-363

hsa-miR-363-3p

hsa-miR-367

hsa-miR-101

hsa-miR-101ab

hsa-miR-31

hsa-miR-125a-5p

hsa-miR-125b-5p

hsa-miR-351

hsa-miR-670

hsa-miR-4319

hsa-miR-10abc

hsa-miR-10a-5p

hsa-miR-455-5p

hsa-miR-128

hsa-miR-128ab

hsa-miR-133abc

hsa-miR-143

hsa-miR-1721

hsa-miR-4770

hsa-miR-204

hsa-miR-204b

hsa-miR-211

hsa-miR-122

hsa-miR-122a

hsa-miR-1352

hsa-miR-24

hsa-miR-24ab

hsa-miR-24-3p

hsa-miR-29abcd

hsa-miR-103a

hsa-miR-107

hsa-miR-107ab

hsa-miR-124

hsa-miR-124ab

hsa-miR-506

hsa-miR-33ab

hsa-miR-33-5p

hsa-miR-490-3p

hsa-miR-137

hsa-miR-137ab

hsa-miR-144

hsa-let-7

hsa-let-98

hsa-let-4458

hsa-let-4500

hsa-miR-26ab

hsa-miR-1297

hsa-miR-4465

hsa-miR-27abc

hsa-miR-27a-3p
