# Supplementary material for: Signatures of tumor microenvironment-related genes and long noncoding RNAs predict poor prognosis in osteosarcoma
Source: PLoS One. 2025 Jul 16;20(7):e0326876. doi: 10.1371/journal.pone.0326876 (PMC12266395; doi:10.1371/journal.pone.0326876)
Supplement: S2 Appendix — (DOCX) [file pone.0326876.s002.docx]

CDK6

CLOCK

DICER1

DYRK2

VCAN

CNNM2

CDK8

CSNK1G3

BAZ2A

AGO1

PURB

NUP50

RUNX1T1

PIK3R1

OGT

BTLA

G3BP2

CALU

CPEB3

CAB39

SLAIN2

ATP13A3

ELK4

FGF2

SALL1

ABL2

C16orf72

SNCG

LCOR

YTHDC1

PPP6C

ATXN1L

TRPS1

DHX33

COPZ1

ZNF385A

MORC2

TP53INP1

STAT3

TRAF6

OSBPL9

TBC1D1

KLF13

ZDHHC9

STOX2

ZSWIM6

ATL2

LIFR

TEF

DOCK3

ARID3A

ARID3B

BTG2

ATXN1

LFNG

ANKRD33B

ZFYVE1

MFSD9

STARD13

DRAM2

C19orf54

C15orf39

TET2

EIF5A2

CDH5

SUV39H1

PLEKHA8

BCL2

ETS1

BCL2L2

HOOK3

FIGN

CDC42EP3

SESN3

NFIB

AGO3

RSBN1

RPS6KA5

NEUROD2

CALM1

TENT4B

SUMO2

GLUL

SHISA9

PTBP2

TAGLN2

CNN2

SP1

MCL1

FOXL2

CPNE3

SUPT16H

KPNA6

FOXC1

TMEM170A

BRWD1

SEMA6D

MMGT1

C8orf58

PPM1A

RNF38

LBR

TNRC6B

NLGN4X

FUT4

SESN2

FNIP1

WEE1

LPIN1

SZRD1

YAP1

SUCO

PHLPP2

NEURL1B

KRAS

DYNLL2

LRATD2

E2F7

PPIF

ZBTB20

TMED5

INO80D

ZNF106

LPCAT1

FOXO1

PLAGL2

MED14

ZFP36L1

ELL2

PSPC1

RAP1B

FOXP4

PEG10

TGFBR3

PSD3

ESR1

KREMEN1

BCL11B

DUS2

RRN3

AURKA

NUP43

SPRYD4

LHFPL2

ESRP1

SLC25A36

NUFIP2

SOX11

KIF5B

KCNC4

ITPRID2

MKNK2

REST

EIF1AD

ANAPC16

CCND2

CREBRF

UBR3

UBN2

AGPAT5

AKIRIN1

EDEM3

MAPK6

ZFAND5

TRIM33

IPO7

YOD1

MIXL1

RASGEF1A

BLCAP

TNKS2

DPP8

MBNL3

SPRED1

POU2F1

ATAD2

SAR1B

GNB5

KLF3

NR2F2

GDF11

MAP1B

MAP3K2

SBNO1

CUL3

SLC22A23

KIAA0513

LCLAT1

ZFYVE26

TAOK1

RAB11FIP1

SAMD12

HMBOX1

CADM2

HIVEP2

ELAVL2

FAM102B

DAZAP2
